# Public perceptions of artificial intelligence in healthcare: ethical concerns and opportunities for patient-centered care

**DOI:** 10.1186/s12910-024-01066-4

**Published:** 2024-06-22

**Authors:** Kaila Witkowski, Ratna B. Dougherty, Stephen R. Neely

**Affiliations:** 1 Florida Atlantic University, Boca Raton, FL USA; 2 University of South Florida, Tampa, FL USA

**Keywords:** Artificial intelligence, Public perception, Patient-centered care, Patient-physician relationship

## Abstract

**Background:**

In an effort to improve the quality of medical care, the philosophy of patient-centered care has become integrated into almost every aspect of the medical community. Despite its widespread acceptance, among patients and practitioners, there are concerns that rapid advancements in artificial intelligence may threaten elements of patient-centered care, such as personal relationships with care providers and patient-driven choices. This study explores the extent to which patients are confident in and comfortable with the use of these technologies when it comes to their own individual care and identifies areas that may align with or threaten elements of patient-centered care.

**Methods:**

An exploratory, mixed-method approach was used to analyze survey data from 600 US-based adults in the State of Florida. The survey was administered through a leading market research provider (August 10–21, 2023), and responses were collected to be representative of the state’s population based on age, gender, race/ethnicity, and political affiliation.

**Results:**

Respondents were more comfortable with the use of AI in health-related tasks that were not associated with doctor-patient relationships, such as scheduling patient appointments or follow-ups (84.2%). Fear of losing the ‘human touch’ associated with doctors was a common theme within qualitative coding, suggesting a potential conflict between the implementation of AI and patient-centered care. In addition, decision self-efficacy was associated with higher levels of comfort with AI, but there were also concerns about losing decision-making control, workforce changes, and cost concerns. A small majority of participants mentioned that AI could be useful for doctors and lead to more equitable care but only when used within limits.

**Conclusion:**

The application of AI in medical care is rapidly advancing, but oversight, regulation, and guidance addressing critical aspects of patient-centered care are lacking. While there is no evidence that AI will undermine patient-physician relationships at this time, there is concern on the part of patients regarding the application of AI within medical care and specifically as it relates to their interaction with physicians. Medical guidance on incorporating AI while adhering to the principles of patient-centered care is needed to clarify how AI will augment medical care.

**Supplementary Information:**

The online version contains supplementary material available at 10.1186/s12910-024-01066-4.

## Introduction

Patient-centered care has been at the forefront of healthcare for over a decade, with scholarship identifying how this critical issue has been a concern for both healthcare organizations and patients [1, 2]. Focusing on the ideals of whole-person care and shared decision-making [3], patient-centered care has improved health outcomes, reduced medical costs, and enhanced patient and physician satisfaction [2, 4, 5]. With the rapid advancement of artificial intelligence (AI) in healthcare, some scholars are concerned that developments in patient-centered care will be overlooked in favor of technological improvements or, more importantly, that the use of AI will directly conflict with the ideals of patient-centered care [3]. While AI has the potential to revolutionize healthcare [6], concerns over how AI is incorporated within the context of patient-centered care need to be addressed to create patient buy-in and ensure the effective adoption of these technologies. Moreover, choices that promote decision self-efficacy and patient perspectives on this emerging technology should be incorporated so that the use of AI in healthcare is beneficial and equitable for all patients [6–9].

This research investigates public perceptions of and attitudes toward AI-enabled healthcare, including perceptions of current levels of comfort with several proposed/active applications of AI for health-related tasks. In our survey, we defined AI-enabled healthcare as the use of computers to imitate or simulate human intelligence related to patient administration, clinical decision support, patient monitoring, and healthcare interventions [10]. Using five common elements of patient-centered care in family medicine [11], this paper analyzed data from a web-based survey of 600 US-based adults from the State of Florida. Using quantitative analysis of multiple-choice questions and qualitative content analysis of open-ended responses, this study sheds light on the role of decision self-efficacy in AI-enabled healthcare and highlights potential concerns and opportunities for incorporating AI in patient-centered care from the viewpoints of public respondents.

### Elements of patient-centered care

Patient-centered care has become an integral part of the American healthcare system. Focusing on the ethical and moral implications of involving patients within the healthcare process on their own terms [1], the implementation of patient-centered care has involved a paradigm shift for doctors and medical professionals to be more empathetic and collaborative with patients [1, 3, 12, 13]. While definitions of patient-centered care can vary significantly across medical professions and healthcare contexts [11, 12, 14], there are often core elements that are considered integral parts of patient-centered care. According to Mead and Bower [14], these elements can include the therapeutic alliance, doctor-as-person, shared power and responsibility, patient-as-person, and the biopsychosocial perspective (i.e., the whole person).

The therapeutic alliance, doctor-as-person, and shared power and responsibility focus on the patient-physician relationship. Within patient-centered care, doctors place value on the quality of their relationships with patients and believe that this relationship can influence medical adherence and self-efficacy (i.e., therapeutic alliance) [14–16]. Patients are considered experts in their experiences and are provided with all the information necessary to make informed decisions regarding their care [3, 14, 17]. Therefore, doctors consider patients to be equal partners in the decisions that affect their care (i.e., shared power) and focus on not only providing information to patients but try to do so in a way that is respectful, empathetic, caring, and sensitive to the experiences, beliefs, and concerns of patients (i.e., doctor-as-person) [14, 18].

Patient-as-person and the biopsychosocial perspective consider the personal meanings associated with symptoms, illnesses, and potential interventions (i.e., patient-as-person) and how these individual interpretations can interact with the biological, psychological, and social environments in which they occur (e.g., culture, the economy, etc.) [14]. Thus, an important consideration for doctors within patient-centered care is to take into account the values, beliefs, and preferences of patients and to design medical care around these preferences [3, 19]. While some scholars note patient preference and ability to remain actively involved in care varies [19], others highlight the important role of actively engaging patients to learn about their preferences and empower them to participate in healthcare decisions (i.e., increase levels of self-efficacy) [15, 16, 20]. While patient-centered care has been a staple in evaluating the quality of care for the last decade [1], there are uncertainties about how AI will impact patient-centered care and, more importantly, a lack of understanding regarding how patients feel about the use of AI in their own care.

### Advancements in the use of AI in healthcare

AI has been called the ‘fourth industrial revolution’ [6, pg. 1] and is anticipated to bring a new frontier for the medical community [21]. Deep learning networks and machine learning algorithms can use data from medical records, clinical registries, and medical journals to anticipate potential patient outcomes [3, 22, 23]. While acknowledging the technical limitations of these tools, many have suggested that AI-enabled healthcare may help to increase equity in health outcomes, reduce diagnostic errors, improve treatment protocols, and even offset increasing labor shortages among health practitioners [10, 24–27]. Some scholars even suggest that AI can improve patient autonomy and self-efficacy by providing patients access to their data [28] or even suggesting treatment options that patients with similar diagnoses made [29].

While these potential outcomes are impressive, the rapid development of this technology is set to outpace physician knowledge in the near future and may even displace the work of some medical professionals [23]. While such advancements could improve the accuracy of diagnoses, they also raise ethical concerns for physicians and implementation concerns for patients. For physicians, advancing technology could bring ethical requirements to consult AI before making decisions [3], thereby limiting professional autonomy. It may also create barriers to patient-physician relationships as physicians may have to explain the rationale behind a diagnosis that they may not fully understand or make themselves [3].

For patients, some scholars have found that there are concerns about how AI is designed and whether these systems are trustworthy [7, 30]. For example, a study by Hallowell and colleagues [30] on the use of AI to diagnose rare diseases found that patients were concerned about the accuracy of these tools and stressed the importance of using AI within a trusted patient-doctor relationship. Another study by Dlugatch and colleagues [7] found that within a labor and delivery setting, birth mothers were concerned about the potential for bias within this technology, raising concerns over representativeness and private AI developers. Studies conducted outside of the U.S. context have also found that trust/acceptance in AI may be higher in some specializations (such as dermatology) than others (i.e., radiology and surgery) [31]. Moreover, evidence has suggested that patients are wary of AI insofar as they perceive it to threaten personal interactions with human practitioners [32]. Although limited to specific medical settings, these studies underscore conversations within the medical community about how to design ethical AI systems that can account for bias and the potential motives of developers while also integrating patients’ values [6, 7, 30, 33].

Despite emerging research on this topic, ethical guidelines and regulation of AI in medical settings have lagged behind advancing technology [21, 34], raising concerns within the medical community on how AI should be implemented in practice. This concern was highlighted by the former WHO Director-General, who stated, “As so often happens, the speed of technological advances has outpaced our ability to reflect these advances in sound public policies and address a number of ethical dilemmas” [21, para.4]. Moreover, perspectives from key stakeholders, including public preferences on this technology, are often missing from these conversations [6, 7]. Without an understanding of public attitudes and acceptance of AI, real-life implementation may face challenges and threaten patient outcomes. This was noted by Yakar and colleagues [31] who found that little attention was paid to the public toward the deployment of “these systems into the practice of patient care” (p. 374). A more recent study conducted by the Pew Research Center (PRC) in February of 2023 [35] found that most Americans report “significant discomfort… with the idea of AI being used in their own health care”. However, this study focused only on a generic and limited range of proposed AI applications.

In this study, we seek to build on work done by Pew [35] and others in order to better understand Americans’ perceptions of and attitudes toward AI-enabled healthcare, including their current levels of comfort with several proposed/active applications of AI for health-related tasks. We report results from a sample of 600 U.S.-based adults using an exploratory, mixed-methods approach. The results are discussed below in the context of patient-centered care in the hopes that more patient and public concerns regarding this technology are incorporated into medical standards.

## Methods

A web-based survey of 600 US-based adults from the State of Florida was conducted (August 10 to August 21, 2023) through Prodege MR, an industry-leading market research provider. The survey was funded by the Florida Center for Cybersecurity. Participants were recruited using a stratified quota sampling approach to ensure that the sample was representative of the state’s population based on gender, age, race, ethnicity, and political affiliation. Quotas were determined (and stratified by region of the state) based on data from the U.S. Census Bureau and Florida’s Office of Economic and Demographic Research. In order to ensure the protection of human subjects, the study was reviewed and approved by the University of South Florida’s Institutional Review Board (IRB Study #005962). Based on the sample size, responses to the survey are reported with a 95% confidence level and a margin of error +/- 4. Table 1 provides a demographic summary of the survey respondents relative to the state’s population parameters.

We began by presenting the following prompt to participants which provides a general definition of AI:

In recent years, there have been significant developments in the area of “Artificial Intelligence”, which refers to the creation and programming of machines that can process information and complete tasks at a level on par with humans.

**Table 1:** Sample comparison

	Sample Demographics	Florida Demographics*
*Gender*		
Female	51.00%	51.10%
Male	48.70%	48.90%
Non-Binary/Other	0.30%	-
*Age*		
18–24	10.70%	10.80%
25–44	32.20%	31.20%
45–64	31.80%	32.40%
65+	25.30%	25.60%
*Race*		
Black/African American	17.20%	16.90%
White/Caucasian	71.80%	77.30%
Other	11.00%	5.80%
*Ethnicity*		
Hispanic	29.00%	26.40%
Non-Hispanic	71.00%	73.60%
*Education*		
Less than 4 Year Degree	67.20%	69.50%
4 Year Degree (or higher)	32.80%	30.50%
*Political Affiliation (registered voters only, n = 524)*		
Democrat	34.40%	36.20%
Independent / Other	29.00%	28.10%
Republican	36.60%	35.70%
Region		
Panhandle	7.20%	7.20%
Northeast Florida	13.80%	12.40%
Central Florida	25.20%	25.50%
West Coast	23.00%	21.90%
Southeast Florida	30.80%	32.90%

*Gender, race, ethnicity, and region quotas based on U.S. Census Bureau’s Population Estimates Program (PEP): https://www.census.gov/quickfacts‌/FL Age quotas based on Florida’s Office of Economic and Demographic Research (EDR): https://www.edr.state.fl.us/Content/population-demographics/‌data/index-floridaproducts.cfm_Political Affiliation quotas based on Florida Division of Elections https://www.dos.myflorida.com/elections/data-statistics/voter-registration-statistics/voter-registration-reportsxlsx/voter-registration-by-party-affiliation/

Survey participants were then asked to indicate their level of *comfort* with AI being utilized for a range of tasks in their “own personal healthcare.” In constructing the survey instrument, we emphasized four distinct areas of application identified by Reddy and colleagues [10]. These included (1) patient administration, (2) clinical decision support, (3) patient monitoring, and (4) healthcare interventions. From the survey results, we created an *AI_Comfort_Scale* (see Table 2) by coding and summing responses to questions related to confidence in AI-enabled healthcare interventions, where “very comfortable” = 4 and “not at all comfortable” = 1 (potential range 8–32, X̄ = 20.223, σ = 5.805). Thus, the *AI_Comfort_Scale* was informed by previous studies on the application of AI in healthcare [10], but the questions were developed by the researchers associated with this study. A summary of the measured items and psychometric properties for this scale is available in Table 3.

The *AI_Comfort_Scale* was regressed (Table 4) using a simple OLS technique against standard demographic categories, political affiliation, and an abbreviated, 6-item version of the *Decision Self-Efficacy Scale* (potential range 6–24, X̄ = 20.715, σ = 2.918), which measures an individual’s confidence in their own abilities to make personal medical decisions [37, 38]. This latter variable was included to account for the possibility that those who feel more confident in their own medical decision-making ability will likely feel less threatened by the presence of AI-enabled options.

Additionally, respondents were encouraged to share their general attitudes regarding AI-enabled healthcare through an open-ended prompt: *In a few sentences, please tell us how you feel about the use of artificial intelligence in healthcare settings*. This approach was selected in order to support a mixed-methods analysis, including an empirical summary of prevailing levels of comfort with AI-enabled healthcare, as well as a textual analysis of the motivations underlying these attitudes. Responses were analyzed using basic descriptive techniques, OLS regression, and qualitative content analysis.

**Fig. 1:**
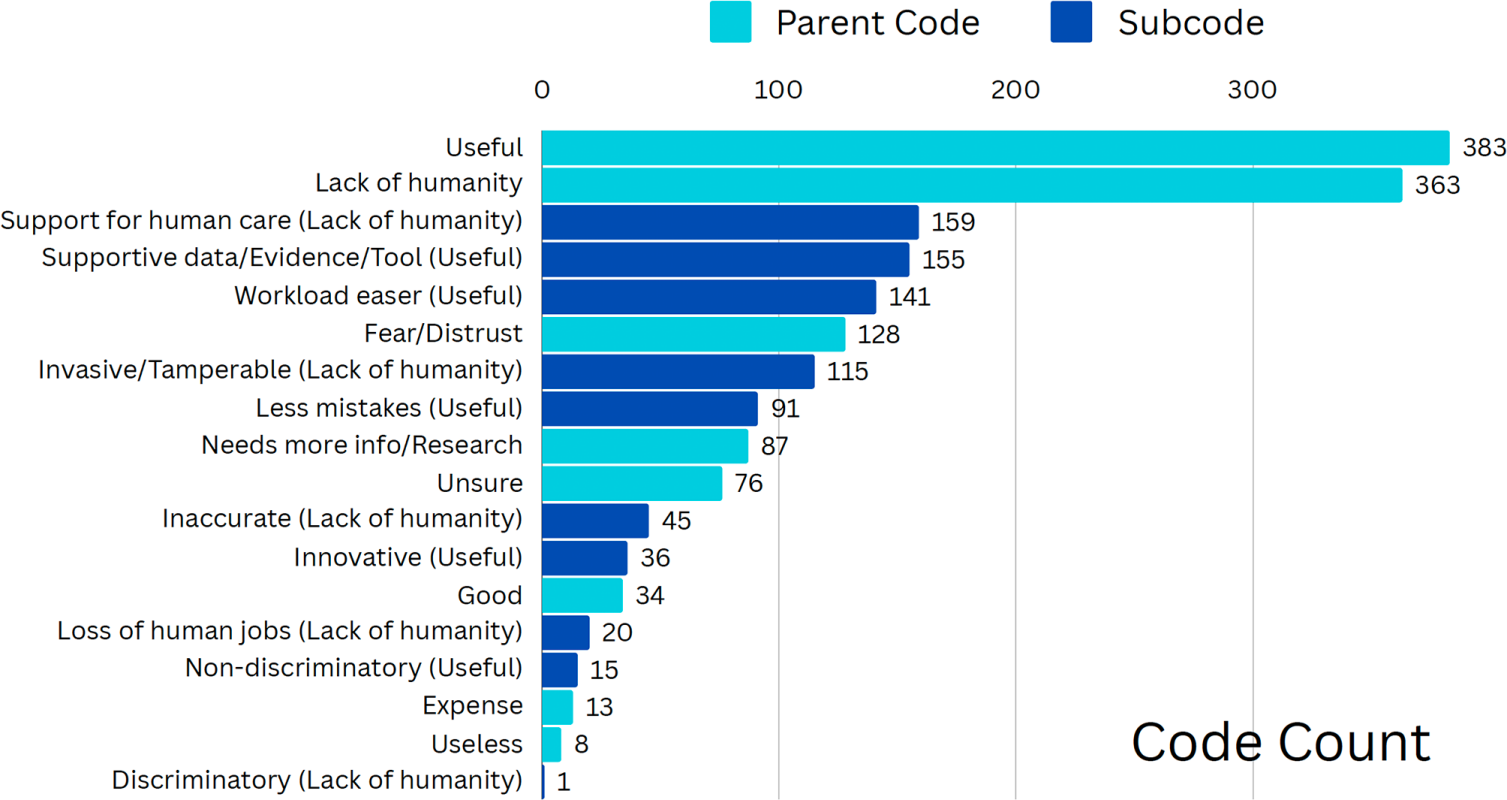
Qualitative coding scheme Note: Codes are not mutually exclusive. Parent codes are indicated in light blue. Subcodes are indicated in dark blue and include parent nodes in parentheses

The qualitative content analysis was conducted in Dedoose using both deductive and inductive methods. Following Braun and Clarke [36] and Klein and colleagues [37], one researcher read through the first twenty responses to get an idea of the general ‘feelings’ that emerged from the responses and then compared them to a priori research. From there, the researcher built three initial codes: 1) *needed more information/research on the topic*, 2) *lack of humanity*, and 3) *usefulness* of AI in healthcare. As the researcher went through each response, additional codes and child codes (reflected under these main codes) were built. The final coding scheme is presented in Fig. 1.

At the end of the coding, there were 586 viable responses. A critical part of the method included determining the association of keywords within specific codes for consistency. For instance, if a participant mentioned AI’s ability to “help” healthcare workers, the response would be coded as *workload easer* (child code) and *useful* (parent code). In another example, if a participant noted they didn’t “trust” AI, or were “uncomfortable” with it, the code attached to those comments was *fear/distrust*. Therefore, our analysis contains overlapping codes, which provide a critical snapshot into the thoughts and concerns respondents have about AI’s use in medical settings.

## Findings

### Preferences on AI in the therapeutic alliance and doctor-as-a-person elements of patient-centered care

While roughly half of the survey participants (50.2%, 301/600) agreed that AI would improve patient outcomes, a majority were more comfortable with AI being used for tasks unrelated to the therapeutic alliance (Table 2). For example, 84.2% (505/600) indicated that they were comfortable with the use of AI for administrative tasks like scheduling patient appointments and follow-ups, while 60.7% (364/600) indicated that they would be comfortable with AI being used to enter intake data (such as symptoms and medical histories). However, respondents were somewhat more divided when it came to the use of AI for clinical decision support and patient monitoring. For example, 57.3% indicated that they would be comfortable with the use of AI to interpret medical imaging (344/600), while 52% were comfortable with the use of AI to predict future medical conditions (312/600).

Roughly half of the survey participants also indicated that they would be comfortable with the use of AI to assist doctors in making their diagnosis (298/600), while 44.7% said they would be comfortable with AI recommending medication/treatment plans (268/600). Reported levels of comfort were lowest when it came to healthcare interventions. Only a third of respondents (33.7%, 202/600) indicated that they would be comfortable with AI being used to administer prescribed medications, while 46.2% (277/600) said that they would be comfortable with the use of AI (such as surgical robots) to assist doctors in their surgical procedures.

When providing open-ended feedback, a portion of participants emphasized the concerns they had with the use of AI in a healthcare setting. For instance, 73 people (12%) noted the *fear/distrust* they have for the use of AI in their care, indicating that they might be “uncomfortable” with AI providing treatment and diagnosis independently of doctors (e.g., Participants 319, 354, 241, 89, 49). Participants also mentioned AI’s *lack of humanity* in the open-ended feedback, emphasizing concerns that AI could not provide a “human touch” (178 responses – 30%). For example, one participant stated, “I don’t think it is ideal to completely rely on computers, especially because a big part of healthcare is human interaction” (Participant 321). Other participants mentioned concerns that AI lacked empathy, judgment, respect, close contact, and nonstatistical-based decision-making—all qualities important in the doctor-as-person element of patient-centered care. These open-ended responses highlighted a few concerns that some participants had regarding the use of AI in healthcare.

However, 65% of respondents also highlighted AI’s potential usefulness (i.e., *useful* code) to support doctors within the therapeutic alliance. For example, 19% of respondents mentioned that AI could serve as a *supportive data/evidence/tool* (70 responses) for human healthcare providers. Responses ranged from AI’s “ability to be a great asset in diagnostic evaluations” (Participant 148) to “a tool or a second, third or fourth opinion” (Participant 547). 18% of participants also noted AI’s ability to serve as a *workload easer* (68 responses). In fact, one participant stated, “I can see how AI can improve efficiency in the office space” (Participant 418), with others echoing this sentiment on AI’s potential use to pull patient history and perform vitals, make diagnosis suggestions, and assist with intake screening or scheduling patient appointments (e.g., Participant 510, 524, 277, 246, 144, 45, 378).

**Table 2:** Patient perceptions of AI-enabled healthcare applications (as % of row total)

*Thinking about your own personal healthcare, how comfortable would you be if AI were used for each of the following tasks/purposes.*	**Very Comfortable**	**Somewhat Comfortable**	**Not Very Comfortable**	**Not at All Comfortable**
To collect and enter patient intake data (such as symptoms and medical histories)	21.8	38.8	26.7	12.7
To assist doctors in making a diagnosis	12.8	36.8	29.0	21.3
To schedule patient appointments and follow-ups	40.7	43.5	9.5	6.3
To predict what future medical conditions patients might develop	16.0	36.0	29.7	18.3
To recommend medications and treatment plans for patients	12.3	32.3	35.3	20.0
To read and interpret medical imaging, such as X-rays and radiology images	16.3	41.0	26.3	16.3
To administer prescribed medications	9.8	23.8	37.0	29.3
To assist doctors in conducting surgical procedures (including the use of surgical robots)	12.5	33.7	29.8	24.0

Usf/fau health policy and administration survey, 2023

**Table 3:** Decision self-efficacy scale (as % of row total)

*The items below include some things involved in making informed medical choices, such as whether or not to take a vaccine. For each item, please indicate how confident you feel in your ability to do these things*:	Not at All Confident (1)	Not Very Confident (2)	Somewhat Confident (3)	Very Confident (4)
Get the facts about the medical choices available to me	1.2	4.7	40.2	54.0
Get the facts about the risks and side effects of each choice	1.8	8.8	39.3	50.0
Express my concerns about each choice	1.0	7.7	37.3	54.0
Figure out the choice that best suits me	0.8	6.0	39.0	54.2
Handle unwanted pressure from others in making my choice	1.8	10.7	31.2	56.3
Delay my decision if I feel I need more time	0.8	7.2	29.0	63.0

USF/FAU health policy and administration survey, 2023, α = 0.802; *n* = 600; AIC = 0.189

Overall, survey results supported the use of AI within the therapeutic alliance under healthcare providers’ guidance. While there were some respondents who did not trust AI (12%) and noted it was not good for the future (7%), several respondents in this study (21%) believed that AI does have the ability to be beneficial within the doctor-patient relationships, such as providing support with “split-second decisions [that also require] a human touch” (Participant 435) or easing the workload with simple tasks like diagnostics.

### Preferences on AI in patient-as-person and the biopsychosocial elements of patient-centered care

As shown in Table 4, those with higher rates of Decision Self-Efficacy were more likely to express confidence in AI-enabled healthcare applications, though the effect size was relatively small. A one-unit increase in decision self-efficacy was associated with a 0.324 increase in AI confidence, *ceteris paribus*, meaning that roughly a one standard deviation increase in decision self-efficacy (σ = 2.918) would be associated with a 1-point increase in AI confidence. Conversely, age was negatively associated with AI confidence (β = -0.065), suggesting that younger respondents are more amenable to AI-enabled interventions in their individual care. Females were significantly and substantially less comfortable with the use of AI in their personal healthcare (β = -3.036) when compared to males. Education was also a significant factor in respondent’s level of comfort with the use of AI in healthcare settings. Those who held a 4-year college degree (or higher) were significantly more comfortable with AI-enabled interventions (β = 1.430) than their counterparts, *ceteris paribus*.

Within the open-ended responses, there were significant concerns about the *lack of humanity* associated with AI and how this would impact not only participants’ understanding of their own health but also the impact this would have on their lives in general. Overall, 62% of participants (363) had comments relating to AI’s *lack of humanity.* While common co-occurrences with this code included its potential to be *useful* (65 responses – 18%), these comments were typically prefaced with concerns about AI replacing their own decision-making. For example, Participant 535 stated, “for appointments and reading scans, it wouldn’t bother me. I don’t want AI making my medical decisions.”

There were mixed results on how AI advancements would impact different aspects of their lives, including workforce and economic concerns. For example, 13% of respondents (49 responses) mentioned AI’s *invasive/tamper-prone nature* made it difficult to encourage a larger role in healthcare. One participant noted, “It is a machine programmed by a human and could be dangerous” (Participant 498). Another noted that “computers are taking over while people don’t realize that they are going to replace humans. Never mind computers can get viruses, they glitch and they crash” (Participant 78). A small number of responses (less than 2%) pointed out that expense was a consideration—AI could make healthcare cheaper, or it could make it more costly. One participant called attention to the fact that AI “will be helpful as long as [it is] affordable and cover[ed] by insurance” (Participant 496). Another questioned, “AI could probably be more capable than any human. But would that make healthcare more expensive than it already is?” (Participant 229). While there was a concern about the invasive nature of AI, 3% of respondents mentioned that it could improve healthcare by making it less discriminatory. One participant clarified, “If we have thoughtfully made AI with a large enough data set, we can potentially eliminate some of the problems we face in medicine. I have had so many doctors ignore my health complaints” (Participant 387).

**Table 4:** OLS regression on AI healthcare comfort scale

	β	s.e.	*p*
Decision Self-Efficacy Scale	0.324	0.079	0.000
Age	-0.065	0.015	0.000
*Gender*			
Male (ref. cat.)	-	-	-
Female	-3.036	0.466	0.000
Other/Non-Binary	-3.708	3.949	0.348
*Race*			
White (ref. cat.)	-	-	-
Black/African American	0.343	0.669	0.609
Other	0.646	0.766	0.400
Hispanic (1 = yes)	-0.046	0.549	0.934
*Political Affiliation*			
Democrats (ref. cat.)	-	-	-
Independents	-0.475	0.621	0.445
Republicans	-0.752	0.621	0.227
Non-Voters	-0.615	0.775	0.428
4-Year College Degree (1 = yes)	1.430	0.487	0.003
Constant	17.029	1.795	0.000
*F*	7.300	-	0.000
R2	0.120	-	-

**Fig. 2:**
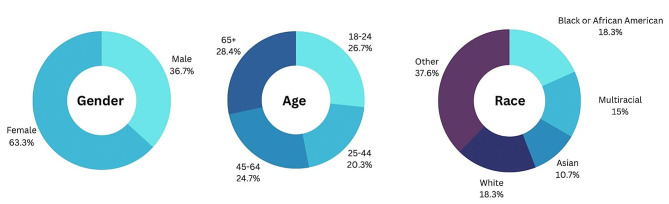
Fear/distrust code by gender, age and race

When considering patient characteristics and the broader social environment, there were slight differences across age groups, party affiliation, household income and education within the open-ended responses. Interestingly, women were more likely than men to mention *fear/distrust* in the use of AI in health-related interventions (see Fig. 2). Respondents identifying as Asian had the least *fear/distrust* (10.7%), while those identifying as Other had the most (37.6%). Additionally, there was less *fear/distrust* noted in the participants’ responses as income increased.

There were also slight differences in the classification of AI across age groups. For example, respondents in the 18-24-year-old age range were more likely (38.9%) to classify AI as *invasive/tamper-prone*. Meanwhile, participants in the 65 and older group were the least likely to note AI’s potential to be *useful* in healthcare (19%). However, there were fewer discrepancies across age groups for the *fear/distrust* code (See Fig. 2), with percentages ranging from 20.3% for ages 25–44 to 28.4% for ages 65 and over. Finally, there were differences across political parties, with Republicans (30.2%) and Independents (27.2%) more likely to identify AI as *invasive/tamper-prone* as opposed to Democrats (10.2%). However, these differences were less noticeable when analyzing the *useful* code, with 35% of Democrats, 31.6% of Independents, and 23.8% of Republicans identifying the potential for AI to be *useful* in healthcare.

## Discussion

This study examined public preferences and comfortability with the use of AI in some aspects of patient-centered care. Using data from a representative survey of adults in the State of Florida (*N* = 600), we found several challenges to address as well as opportunities to explore when implementing AI within a healthcare setting. First, patients were more comfortable with the use of AI outside of the therapeutic alliance, with the majority of respondents (84.2%) expressing greater comfort with the use of AI to schedule patient appointments or follow-ups. Supplemental qualitative data suggested that the fear of losing a “human touch” with doctors was a potential driving force for these responses. These results highlight a potential concern that AI will impact the therapeutic alliance and potentially reduce the positive relational elements (i.e., empathy, understanding, etc.) present within the doctor-as-a-person elements of patient-centered care.

Similar to emerging research on the use of AI in healthcare, the results of this study underscore restrictions on the use of AI and the need to outline processes that complement the therapeutic alliance instead of replacing it [29, 38]. Previous research on patient-centered care has found that physician communication plays an essential role in identifying patient preferences and engaging patients to participate in their own healthcare decisions [15, 16, 38]. According to Epstein and colleagues [18], this style of communication requires doctors to go “well beyond providing just facts and figures… [using this] approach, the clinician frames and tailors information in response to an understanding of a patient’s concerns, beliefs and expectations” (p. 1491). If AI were used within the therapeutic alliance, some scholars believe that aspects of doctor-patient relationships would suffer [38], particularly in how doctors explain the reasoning behind a diagnosis and treatment recommendations [3]. To overcome these concerns, some scholars recommend limiting the use of AI in the decision-making process [3], providing training to doctors on how to use AI and explain outputs to patients [30, 38], or having doctors maintain control over its implementation, including the ability to override incorrect diagnoses [6].

Second, higher rates of decision self-efficacy were associated with greater confidence in the use of AI in medical care. Self-efficacy is an essential tool in patient-centered care, crossing over the elements of patient-as-a-person, the biopsychosocial perspective, the therapeutic alliance, and shared power and responsibility. Specifically, patients require self-efficacy and autonomy in order to communicate their personal meanings, values, and preferences to their healthcare provider [39]. Therefore, the results of this study may show that patients who are more comfortable with communicating their preferences and more active in their care may be more willing to accept AI within this process. However, there was limited qualitative data to explain this phenomenon, and more research is needed to parse out the relationship between self-efficacy and AI-enabled healthcare acceptance. Despite these limitations, the results may suggest that including AI within informed consent procedures and specifically outlining the harms and benefits before using AI with patients [38] may help them feel a higher sense of self-efficacy and control over which aspects of AI they want to use within their own care.

Third, there were concerns about how AI would impact respondents’ lives in general, highlighting potential concerns within the biopsychosocial element of patient-centered care. These concerns include fears that AI would replace humans (workforce concerns), the impact of AI on healthcare costs (economic concerns), and the reliability of human-designed AI systems. While many of these concerns represent overarching fears about the incorporation of AI within daily human life, they can impact patient acceptance and comfort with the use of this technology in their own care. Therefore, patient concerns regarding the implementation of AI in medical care as well as its impact on society should be considered to ensure equitable and efficient implementation. Some of these fears could be addressed by establishing mechanisms for transparency and accountability [30]. Scholars have consistently highlighted the need to develop regulation and oversight mechanisms that address AI’s implementation in medical care [6, 34]. These regulation and oversight mechanisms should account for issues related to patient choice and consent [34], transparency and accountability for private companies developing AI tools [7], guidance on how to design ethical AI systems [33] and should establish who has access to confidential data [6, 34]. Recommendations for how to adapt AI to patient-centered care should also be developed, along with specific guidance for doctors on implementing AI within different medical settings.

Finally, a significant portion of the open-ended responses mentioned the benefits of including AI in patient care. These benefits included being a supportive tool for doctors, the potential to reduce healthcare costs, and the possibility of creating a more equitable healthcare process, particularly as it relates to patient choice and healthcare concerns. This may identify a potential avenue for AI to assist practitioners in understanding patient concerns (i.e., patient-as-person), void of personal or professional biases. However, other scholars have noted AI’s potential to inappropriately ‘nudge’ participants to a specific behavior, even acknowledging potential paternalistic uses of this technology towards minority populations [38]. This further justifies a need to incorporate patient consent within the use and application of AI in medical care [6, 34, 38] and the need to find ways to incorporate patient choices, values, and beliefs within the development of AI tools [7].

Based on the results of our study, we have several recommendations for how AI can be incorporated within patient-centered care. First, we recommend that doctors and healthcare staff consider a patient’s individual comfort level with the use of AI within their care. This can be incorporated into intake procedures by including a consent form on the use of AI at this healthcare facility. This consent form should be written in plain language with clear descriptions on the optional use as well as an outline of the risks and benefits of using AI. Second, we recommend that healthcare agencies interested in incorporating AI at their facilities start by implementing AI outside of the therapeutic alliance. An example of an area our respondents indicated that they were comfortable using AI was to schedule patient appointments or follow-ups. Third, if it is used within the therapeutic alliance, we recommend that AI be utilized as a supportive tool in conjunction with doctors’ experiences and knowledge. We also encourage doctors to be transparent about the use of AI in assisting with patient diagnoses and care. While we acknowledge the potential efficiency of using AI, patient comfort and consent should be incorporated by allowing patients to have choices in how AI is incorporated and be informed of its use.

### Limitations

There are several limitations associated with this study. First, the survey was designed to capture general preferences and levels of comfort with the use of AI for health-related tasks. As such, specific elements of patient-centered care were not as prevalent as others, including shared power and responsibility. Future research could focus on each aspect of Mead and Bower’s [14] conceptualization of patient-centered care to get a more in-depth understanding of patient preferences within each element. Second, we used a supplemental open-ended question to gain insight into why respondents selected specific answers about AI-related healthcare tasks. Future research could conduct in-depth follow-up interviews to gain more insight into the nuances associated with this topic. Third, the study is limited to public perceptions of AI within the context of Florida. Future research should examine actual patient’s experiences with AI and could compare our results with public perceptions in different states and from the perspective of healthcare professionals. Fourth, there are numerous ways to measure self-efficacy and future research could use alternative measures, such as Lorig and colleagues’ self-efficacy scale [40] to verify our results. Fifth, the inclusion of additional controls may help us better understand the factors that determine how comfortable patients are with the adoption of AI in healthcare settings, as well as the primary barriers to acceptance/adoption. Among these may be measures of the individual’s socio-economic status and health insurance status, as well as their own use of and comfort with advanced/emerging technologies. Notably, prior studies have found personal technology usage and perceived technological self-efficacy to be key predictors of technology acceptance [42, 43]. Future studies can incorporate these controls to further validate results. Lastly, this study utilized a single coder for the analysis of the open-ended responses and having at least two coders for this could have strengthened the reliability of the results. As future research delves into this subject more with greater focus on the rich, textual analysis that is possible from open-ended questions, this can be remedied.

## Conclusion

The medical field is experiencing a period of rapid technological advancement due to the increasing use of AI in medical care. While there are significant benefits to the use of AI, patient preferences, concerns, and fears about the use of AI within their own healthcare need to be addressed to ensure that aspects of patient-centered care are not overlooked. While this study identifies some areas of patient-centered care that can benefit from AI, there are significant public concerns that need to be addressed, including how AI will be used within the patient-physician relationship and in what ways AI will impact patient choice and consent. Government regulation and oversight are needed to address implementation concerns, and medical recommendations are needed to identify how AI should be implemented within patient-centered care.

## Electronic supplementary material

Below is the link to the electronic supplementary material.

Supplementary Material 1

## Data Availability

Data was publicly released on September 6, 2023 and is available on the University of South Florida’s webpage: https://www.usf.edu/arts-sciences/departments/public-affairs/documents/news-items/spa-florida-public-health-policy-survey-results-2023.pdf. Qualitative data is available upon request. The survey guide is available in the supplementary file section.
