# Supplementary material for: Public perceptions of artificial intelligence in healthcare: ethical concerns and opportunities for patient-centered care
Source: BMC Med Ethics. 2024 Jun 22;25:74. doi: 10.1186/s12910-024-01066-4 (PMC11193174; doi:10.1186/s12910-024-01066-4)
Supplement: Supplementary file 1 — Supplementary Material 1 [file 12910_2024_1066_MOESM1_ESM.pdf]

qualtrics XM

Florida Health Policy and Administration Survey

Decision Self-Efficacy and Artificial Intelligence Survey Guide

---

Decision Self-Efficacy Scale

The items below include some things involved in making informed medical choices, such as whether or not to take a vaccine. For each item, please indicate how confident you feel in your ability to do these things:

	Very Confident (1)	Somewhat Confident (2)	Not Very Confident (3)	Not at All Confident (4)
Get the facts about the medical choices available to me. (1)	○	○	○	○
Get the facts about the risks and side effects of each choice. (2)	○	○	○	○
Express my concerns about each choice. (3)	○	○	○	○
Figure out the choice that best suits me. (4)	○	○	○	○
Handle unwanted pressure from others in making my choice. (5)	○	○	○	○
Delay my decision if I feel I need more time. (6)	○	○	○	○

End of Block: Decision Self-Efficacy Scale

---

qualtrics XM

Artificial Intelligence Questions

In recent years there have been significant developments in the area of "Artificial Intelligence", which refers to the creation and programming of machines that can process information and complete tasks at a level on par with humans.

In this next set of questions we would like to better understand your opinions about Artificial Intelligence (hereafter referred to as "AI").

Thinking about your own personal healthcare, please indicate how comfortable you would be if AI were used for each of the following tasks/purposes:

	Very Comfortable (1)	Somewhat Comfortable (2)	Not Very Comfortable (3)	Not at All Comfortable (4)
To collect and enter patient intake data (such as symptoms and medical histories) (1)	○	○	○	○
To assist doctors in making a diagnosis. (2)	○	○	○	○
To schedule patient appointments and follow-ups. (3)	○	○	○	○
To predict what future medical conditions patients might develop (4)	○	○	○	○
To recommend medications and treatment plans for patients (5)	○	○	○	○
To read and interpret medical imaging, such as X-rays and radiology images (6)	○	○	○	○
To administer prescribed medications (7)	○	○	○	○
To assist doctors in conducting surgical procedures (including the use of surgical robots) (8)	○	○	○	○

In a few sentences, please tell us what your primary concerns are about the use of Artificial Intelligence in healthcare settings.

____

____

____

____

____

End of Block: Artificial Intelligence

qualtrics XM

Demographic Questions

This final set of demographic questions is being collected for statistical purposes only. As noted above, your responses are confidential and anonymous.

What is your gender?

- ○ Male (1)
- ○ Female (2)
- ○ Other/Non-Binary (3)

What is your age?

▼ 18 (1) ... 99 or older (82)

Are you of Hispanic ancestry?

- ○ Yes (1)
- ○ No (2)

Which of the following best describes your race?

- Black or African American (1)
- Asian (2)
- Hawaii Native/Pacific Islander (3)
- Native American/Alaska Native (4)
- White (5)
- Multiracial (6)
- Other (7) ____

What is your highest level of completed education?

- Less Than High School (1)
- High School Diploma or GED (2)
- Some College (no degree) (3)
- Technical/Vocational Degree (4)
- Bachelor's Degree (5)
- Graduate Degree or Higher (6)

qualtrics XM

What is your annual household income?

- Less than $25,000 (1)
- $25,000 - $49,999 (2)
- $50,000 - $74,999 (3)
- $75,000 - $99,999 (4)
- $100,000 - $149,999 (5)
- $150,000 - $199,999 (6)
- $200,000 and above (7)

Are you currently registered to vote in the state of Florida?

- Yes (1)
- No (2)
- Unsure (3)

What is your political party affiliation?

- Democrat (1)
- Independent (2)
- Republican (3)
- Other (4) ____

Which county do you live in?

▼ Alachua (1) ... Washington (67)

End of Block: Demographic Questions
